# Oral Health Inequities among CALD and Non-CALD Older Australians: A Decomposition Analysis

**DOI:** 10.3390/ijerph20156455

**Published:** 2023-07-27

**Authors:** Lisa Jamieson, Gloria Mejia, Liana Luzzi, Xiangqun Ju

**Affiliations:** Australian Research Centre for Population Oral Health (ARCPOH), Adelaide Dental School, University of Adelaide, Adelaide, SA 5005, Australia; gloria.mejia@adelaide.edu.au (G.M.); liana.luzzi@adelaide.edu.au (L.L.); xiangqun.ju@adelaide.edu.au (X.J.)

**Keywords:** untreated dental caries, decomposition, CALD, older Australians

## Abstract

Background: Among Australia’s older population, the burden of oral disease is disproportionality borne by culturally and linguistically diverse (CALD) communities. This study aims to examine changes in untreated decay surfaces (DS) between 2004–2006 and 2017–2018 among older CALD and non-CALD Australians. Methods: Data were sourced Australian national oral health surveys conducted in 2004–2006 and 2017–2018. An Oaxaca–Blinder decomposition analysis was used to assess the contribution of socio-demographics and dental behaviours. Results: A total of 246 CALD and 2853 non-CALD dentate participants aged 60+ years took part in 2004–2006, and 363 and 4278 in 2017–2018, respectively. There were increases in mean DS for both CALD (0.74 to 1.42) and non-CALD (0.72 to 1.50) groups between 2004–2006 and 2017–2018. The decomposition model showed that, for CALD participants in 2004–2006 with untreated DS, 40% of the contribution was from not having dental insurance; nearly three-quarters of the contribution was from last dental visit being over one year ago (72.9%) in 2017–2018. Among non-CALD participants in 2017–2018 with untreated DS, 42.5% of the contribution was from the last dental visit being over one year ago. Conclusions: Our findings suggest that social determinants, including increased access to dental insurance, could mitigate the oral health inequities observed.

## 1. Introduction

Similar to almost all the Organization for Economic Co-operation and Development (OECD) countries, Australia’s population is ageing [1]. By 2021, the proportion of the Australian population aged 65 years or over exceeded the proportion of Australians aged 15 years or less. By 2051, it is predicted that approximately one quarter of the population will be 65 years or older, with around one-third born in a country other than Australia [2]. One product of the ageing of the population is people living for longer with more teeth in their mouths [3]. But the health of these teeth at a population level is not universally shared, with inequities in oral health in older age groups even wider than those observed among younger and middle-aged Australians. For example, in the 2017–2018 National Study of Adult Oral Health, the mean number of decayed tooth surfaces among those aged 35 to 54 years was 1.4, compared to 1.8 among those aged 55 to 74 years [4]. When stratified by regional location, the mean number of decayed tooth surfaces among those aged 35 to 54 years living in a non-metropolitan setting was 1.3, compared to 1.9 among their counterparts aged 55 to 74 years also living in a regional location. There is a positive association between tooth retention and health-related quality of life, with older adults with 20 or fewer teeth having a higher prevalence of functional dependence and disability [5].

Culturally and Linguistically Diverse (CALD) populations in Australia are broadly defined as being born in a country other than Australia and speaking a language other than English in the home [6]. The largest CALD groups include those originally from countries in Asia, including China and India. The cultural and heritage beliefs of caring for the elderly in CALD populations are in stark contrast to more mainstream Australian values, where residential homes for ageing populations are the norm for most non-CALD populations [7]. By contrast, the model for many CALD populations is for ageing family members to continue residing in the family home, with primary care provided by family as opposed to paid workers [8]. Although there are many benefits to having older people looked after in the family home, including better social and mental health outcomes, there are limitations in accessing services essential for healthy older ageing. This includes access to dental care services. As a consequence, older CALD Australians are recognized as having among the highest levels of unmet dental needs in the country [9].

Using data from the 2004–06 National Survey of Adult Oral Health, Mejia and colleagues [10] identified that almost all (90%) Australian adults have experienced dental caries, with this proportion increasing to 97% for those in the lowest income group. The standard index for assessing dental caries experience, the Decayed, Missing, Filled (DMF) index, indicated that the highest inequities were observed in the ‘missing’ (teeth extracted) and ‘decayed’ (untreated dental caries) components—that is, in the provision of dental care (more socially advantaged groups receiving dental restoration, less advantaged groups receiving extractions) and access to that care (able to access dental health providers resulting in restoration/extractions, unable to access care resulting in untreated dental caries). The most common barriers to accessing dental care include cost and location of dental practice, especially for those living in regional or remote locations [11]. Limited access to dental care may result in potentially avoidable hospital admissions, with dental conditions being the second-highest cause of preventable hospitalisations in Australia in 2015–2016 [12]. However, unlike hospital care, public dental services are not universally accessible. State and Territory Governments are primarily responsible for these services, with funding support from the Australian Government through National Partnership Agreements. Approximately 23 percent of the Australian population are eligible for publicly funded dental care [13]. The remainder can only access dental care through the private sector, which are typically located in urban centres. Private dental patients pay out-of-pocket for their dental care, with some of these costs offset for those with dental insurance services.

Australia’s National Oral Health Plan (2015–2024) identified CALD and older populations as priority areas but did not consider how the intersectionality of being both older and CALD might pose challenges that are greater than the sum of its parts [14]. This study aims to examine changes in untreated coronal decay between 2004–2006 and 2017–2018 among CALD and non-CALD Australians aged 60+ years and to estimate the contribution of demographics, socioeconomic position and dental utilization patterns to facilitate more effective and culturally safe dental service provision models for older CALD populations. The hypothesis is that the percentage contribution of each risk factor will differ for CALD and non-CALD groups, and that these differences would increase over time. The findings are likely to have relevance in all countries in which the proportion of older CALD populations is increasing, and who bear a disproportionate burden of adverse oral health outcomes.

## 2. Methods

### 2.1. Study Design and Sample Selection

Data were from two population-based cross-sectional surveys of Australian adult oral health conducted in 2004–2006 (NSAOH 2004–2006) and 2017–2018 (NSAOH 2017–2018) [15,16]. In each survey, representative samples of adults were drawn through a three-stage, stratified sample design within metropolitan and regional areas in each state/territory. The first stage selected a sample of postcodes from all in-scope postcodes in Australia. The second stage selected households within sampled postcodes, with adults aged 15 years and over being randomly selected from each sample household to participate in the final stage. Data were weighted following standard procedures for clustered samples. In this study, only dentate participants aged 60 years or over were included in the analysis. Both NSAOH 2004–2006 and NSAOH 2017–2018 were reviewed and approved by the University of Adelaide Human Research Ethics Committee.

### 2.2. Data Collection

Self-reported information about oral health and related characteristics were collected using a computer-assisted telephone interview (CATI) in 2004–2006, and CATI or online questionnaire in 2017–2018. Information about dental clinical status was collected during standardized oral epidemiological examinations conducted by registered, trained and calibrated oral health professionals. Standard dental clinic lighting conditions were used, with teeth gently wiped with gauze if required to facilitate visual access and examination. Each tooth had five coronal surfaces examined, with dental decay defined as cavitation that had broken or visibly undermined the enamel. All examiners were tested in the field against a gold standard examiner to estimate inter-examiner reliability. The intraclass correlation coefficient (ICC) for the number of teeth present was 1.00 and for the number of decayed tooth surfaces was 0.96.

### 2.3. Variables

CALD status was identified based on English and not the primary language spoken at home. Sociodemographic characteristics included age, sex, residential location, household income, dental insurance and last dental visit. Age was grouped into ’60–69’, ’70–79’, ‘≥80’. Sex was classified as ‘Male’ or ‘Female’. Residential location was categorised into ‘Major city’ or ‘Regional/Remote’. Income was defined by total annual household income, with tax, and categorised as ‘<AUD30,000’, ‘AUD30,000 to <AUD60,000’, ‘AUD60,000 to <AUD100,000’ and ‘AUD100,000+’. Dental insurance was based on a ‘yes’ or ‘no’ response. Last dental visit was derived from the question ‘How long ago did you last see a dental professional about your teeth, dentures or gums?’, with responses dichotomized into ‘Less than one year’ and ‘One year or more’. Country of birth was dichotomized into ‘Australia’ or ‘other’. The outcome measure, mean number of decayed coronal tooth surfaces (DS), was assessed during clinical examination.

### 2.4. Data Analysis

Changes in mean DS between NSAOH 2004–2006 and NSAOH 2017–2018 were evaluated for the whole sample then stratified by CALD status. Oaxaca–Blinder type decomposition analysis was used to assess the contribution of demographic (age, sex, residential location), socioeconomic position (household income, dental insurance) and dental behaviours (last dental visit) to changes in mean DS between 2004–2006 and 2017–2018, in CALD and non-CALD populations. Blinder–Oaxaca decomposition is a statistical method that decomposes the gap in mean outcomes across two groups into a portion that is due to differences in group characteristics and a portion that cannot be explained by such differences [17]. It is a counterfactual analysis that enables investigation into what actually happened and what would have happened in the absence of the intervention. In the current study, it explains the change in the mean DS over time when the CALD or non-CALD individual is set to have, for example, no dental insurance or resides in a non-metropolitan location and the same individual is set to have dental insurance or reside in a metropolitan location. All analyses were conducted using the oxaca command in Stata 13. Weights were used to account for the complex sampling methodology of both surveys.

## 3. Results

A total of 246 CALD dentate participants aged 60+ years took part in 2004–2006 (Table 1). The average age was 68.0 and ranged from 60 to 88 years; about two-thirds (63.2%) were in the 60–69 years age range. Just over half (53%) were female, 85% resided in a major city, almost two-thirds (63.5%) had an annual household income of less than $30,000, 64% did not have dental insurance, 34% last visited a dentist over a year ago and 93% were born overseas. The mean number of teeth was 21. The mean DS was 0.74. Higher levels of untreated dental caries were observed among those with no dental insurance (0.97) and those born overseas (0.81).

A total of 2853 non-CALD dentate participants aged 60+ years took part in 2004–2006. The average age was 70.2 and ranged from 60 to 96 years, more than half (51.8%) were in the 60–69 years age range. Just over half (51%) were female, 63% resided in a major city, 61% had an annual household income of less than $30,000, 54% did not have dental insurance, 33% last visited a dentist over a year ago and 22% were born overseas. The mean number of teeth was nearly 20. The mean DS was 0.72. Higher levels of untreated dental caries were observed among those with low household income (0.92), no dental insurance (0.95) and those born overseas (1.04).

In 2004–06, there were proportionally more CALD than non-CALD participants residing in major cities (85% compared with 63%) and without dental insurance (64% compared with 54%).

A total of 363 CALD dentate participants aged 60+ years took part in 2017–2018. The average age was 70.0 and ranged from 60 to 94 years, over half (55.2%) were in the 60–69 years age range. Just over half (56%) were female, 91% resided in a major city, 55% had an annual household income of between $30 and $60,000, 67% did not have dental insurance, 49% last visited a dentist over a year ago and 93% were been born overseas. The mean number of teeth was 22. The mean DS was 1.42. Higher levels of untreated dental caries were observed among those living in regional and remote Australia (1.49) and those born in Australia (7.00).

A total of 4278 non-CALD dentate participants aged 60+ years took part in 2017–2018. The average age was 70.3 and ranged from 60 to 101 years, and just over half (50.9%) were in the 60–69 years age range. Just over half (51%) were female, 64% resided in a major city, 54% had an annual household income of between $30 and $60,000, 47% did not have dental insurance, 34% last visited a dentist over a year ago and 31% were born overseas. The mean number of teeth was 21. The mean DS was 1.50. Higher levels of untreated dental caries were observed among those living in regional and remote Australia (2.01) and those born in Australia (1.77).

In 2017–2018, there were proportionally more CALD than non-CALD participants residing in major cities (91% compared with 64%), without dental insurance (67% compared with 47%) and who had last visited a dentist 12+ months previously (49% compared with 34%).

When comparing socio-demographic and dental characteristics across the two time points, among CALD participants, in 2017–18, there was a higher proportion in the $30 K to $60 K household income bracket compared with 2004–2006 (56% compared with 24%) and who had last visited a dentist more than twelve months previous (49% vs. 34%). Overall mean DS in the CALD population from 2004–2006 to 2017–2018 had increased from 0.74 to 1.42, with significant increases noted among males (0.47 to 1.88). Among non-CALD participants, in 2017–2018 there was a higher proportion in the ≥$100 K household income group (14% compared with 5%) and with dental insurance (53% compared with 46%). Overall mean DS in the non-CALD population from 2004–2006 to 2017–2018 increased from 0.72 to 1.50, with significant increases noted among males (0.95 to 2.20), those living in regional and remote locations (0.82 to 2.01), those with a household income of <$30 K (0.92 to 2.43), those with no dental insurance (0.95 to 2.12), those who last visited a dentist 12+ months ago (1.38 to 2.70) and those born in Australia (0.62 to 1.77).

The decomposition models (Table 2) demonstrated that, for CALD participants aged 60+ years in 2004–2006 with untreated DS, approximately 40.0% of the contribution was from not having dental insurance, followed by last dental visit being over a year ago (34.4%). Among non-CALD participants aged 60+ years in 2004–2006 with untreated DS, more than four-fifths (80.08%) of the contribution was from low household income. Among CALD participants aged 60+ years in 2017–2018 with untreated DS, nearly three-quarters of the contribution was from last dental visit being over one year ago (72.9%). Among non-CALD participants aged 60+ years in 2017–2018 with untreated DS, 42.5% of the contribution was from last dental visit being over one year ago.

## 4. Discussion

In our analysis, Oaxaca–Blinder type decomposition models were used to quantify how much of the difference in the primary outcome (mean DS) between two groups (CALD, non-CALD) over time (2004–2006 to 2017–2018) is explained by respective differences in the distributions of selected independent variables (age, sex, residential location, household income, dental insurance status, time since last dental visit and country of birth). The hypothesis that the percentage contribution would differ between older CALD and non-CALD Australian adults and that the magnitude of these differences would increase over time proved only partially true. The highest percentage contribution for CALD groups in 2004–2006 was not having dental insurance. Conversely, the highest percentage contribution for non-CALD groups in 2004–2006 was last dental visit over 12 months ago. In 2017–2018, for CALD groups the highest percentage contribution was low household income, while for non-CALD groups it was again last dental visit over 12 months ago. The magnitude of the difference in mean DS did increase over time; for CALD groups by 0.68 and for non-CALD groups by 0.78.

An unanticipated finding was the socio-demographic changes between CALD and non-CALD groups between 2004–2006 and 2017–2018. A higher proportion of older CALD participants in 2017–2018 were female, residing in a major city and had an annual household income of $30–$60,000 compared with their older CALD counterparts in 2004–06. The magnitude of untreated DS differences was also higher among the non-CALD population, despite the proportion of CALD older Australians with dental insurance and last visiting a dentist in the last 12 months being lower than their non-CALD counterparts. These demographic CALD shifts are represented in the Australian population more broadly. For example, through Government initiatives, including the Global Talent visa program, an increasing proportion of newly arrived CALD populations, who frequently arrive with older family members, are highly educated, with an internationally recognised record of professional and academic achievement. This frequently translates into success in completing high-level tertiary education attainment, and in obtaining high paying and permanent employment. Many have benefitted from social advantage and optimal health in their country of origin, which is able to be continued upon migration to Australia.

There are a range of dental insurance schemes in Australia used predominantly by oral health practitioners in the private sector. Many private health insurance plans include dental coverage as an optional extra as part of their ancillary or extras cover. These plans enable policy holders to claim a portion of dental-related costs, including for check-ups, restorations, extractions, crowns and bridges. There are also dental-only schemes, which typically include more comprehensive coverage of complex procedures, such as dental implants. Most plans that include dental in their coverage have benefit limits of the maximum amount able to be claimed for a specific treatment or service within a given timeframe. Dental insurance plans also frequently have a ‘gap’, which requires an out-of-pocket, non-claimable cost borne by the policy holder. Many dental insurance companies also have preferred provider networks. In the 2017–2018 National Study of Adult Oral Health, 51% of the population aged 55–74 years had dental insurance [2]. However, socially disadvantaged older Australians adults are limited in their options regarding dental insurance, and typically rely on care through the public sector. Dental public services in Australia are means-tested, have long wait lists, provide basic (not comprehensive) dental services and often require co-payment. These strategies contribute to increasing inequities, with an accumulative effect of masking demand and facilitating problem-based dental care utilization. This inevitably results in poor dental outcomes, with disease states frequently worsening, spreading to other teeth and requiring more comprehensive care/extractions.

As in many OECD countries, the clinical oral health of older citizens has improved, particularly with retention of natural teeth, but these improvements have not been experienced equally across all older population sub-groups. In the United Kingdom, clinical oral health has improved substantially in the last 50 years, but social inequities have increased [19]. In the United States, a comparison of national survey data of non-institutionalized adults aged 65+ years from 1999 to 2004 and 2011 to 2016 indicated that while mean numbers of missing teeth decreased, inequities in access to restorative care (that is, for rehabilitative services) across income groups had increased [20].

Recommendations to improve the access of older CALD populations to oral health programs include building transcultural dental training into the educational requirements of dental students, developing oral health promotion programs that include culturally and linguistically customized information, and upskilling geriatricians and related auxiliary staff in the importance of oral health in general health and well-being.

Limitations of the study include the definition of CALD being English not primary language spoken at home. Australia’s population includes people who were born overseas, have a parent born overseas and/or speak a variety of languages other than English—as defined by the Australian Bureau of Statistics, these communities are referred to as CALD [21]. A recent Australian Institute of Health and Welfare report on the health of CALD populations discussed the issue of defining CALD [22]—with no definitive conclusion made but instead a suggestion to combine different CALD-defining measures where possible. Numbers did not allow for this in the current study, but a separate analysis was run with CALD defined using ‘place of birth’ with similar results.

## 5. Conclusions

The social determinants with the highest percentage contribution for untreated dental caries among older CALD groups were not having dental insurance and low household income. The highest percentage contribution for older non-CALD groups were last dental visit over 12 months ago. These decomposition findings are relevant for health policy and public health action, as they can indicate which broader determinants could be primarily targeted to influence timely and culturally safe access to dental care for older adult groups in Australia.

## Figures and Tables

**Table 1:** Sample characteristics, mean number of teeth and severity of untreated dental caries between CALD and non-CALD Australians aged ≥60 years between 2004–2006 and 2017–2018 (weighted).

	2004–2006 (n = 3100)								
	Number, Weighted % (95% CI)				Mean Number of Teeth (95%CI)		Mean DS (95% CI)		Ratio ^a^
	CALD		Non-CALD		CALD	Non-CALD	CALD	Non-CALD	
**Total**	245	9.3 (8.0–10.7)	2855	90.7 (89.3–92.0)	20.6 (19.2–21.9)	19.5 (19.2–19.9)	0.74 (0.43–1.07)	0.72 (0.60–0.84)	1.03
**Age group (years)**									
60–69	150	63.2 (56.0–70.4)	1556	51.8 (49.7–56.3)	21.6 (19.9–23.3)	20.8 (20.3–21.3)	0.59 (0.28–0.89)	0.69 (0.52–0.86)	0.86
70–79	79	29.9 (23.2–36.6)	933	34.6 (32.5–36.7)	19.4 (17.0–21.8)	18.3 (17.6–19.0)	1.24 (0.44–2.04)	0.81 (0.60–1.02)	1.53
≥ 80	16	6.9 (3.4–11.7)	366	13.5 (11.6–15.0)	15.0 (8.5–21.5)	17.7 (16.3–19.2)	0.03 (−0.44, 0.50)	0.54 (0.31–0.78)	0.06
**Sex**									
Male	102	47.2 (39.6–54.8)	1215	49.2 (47.0–51.4)	20.9 (19.1–22.8)	19.4 (18.8–20.0)	0.47 (0.14–0.81)	0.95 (0.75–1.15)	0.49
Female	143	52.8 (45.2–60.4)	1640	50.8 (48.6–53.0)	20.2 (18.2–22.2)	19.7 (19.2–20.2)	0.99 (0.47–1.52)	0.49 (0.36–0.63)	2.02
**Residential location**									
Regional/remote	52	**14.8 (9.4–20.2)**	1209	**36.6 (34.6–38.7)**	17.6 (14.3–21.0)	18.4 (17.8–19.0)	1.40 (0.03–2.77)	0.82 (0.65–1.04)	1.71
Major city	193	**85.2 (79.8–90.6)**	1646	**63.4 (61.3–65.4)**	21.1 (19.7–22.6)	20.2 (19.7–20.7)	0.65 (0.36–0.86)	0.64 (0.48–0.79)	1.02
**Household income**									
<$30 K	147	63.5 (55.9–71.1)	1660	61.2 (59.0–63.5)	19.1 (17.3–21.0)	17.9 (17.4–18.4)	0.88 (0.41–1.34)	0.92 (0.74–1.12)	0.96
$30 K to <$60 K	52	24.4 (17.7–31.0)	622	24.6 (22.6–26.6)	21.8 (19.5–24.1)	22.2 (21.5–22.8)	0.73 (0.23–1.25)	0.41 (0.27–0.54)	1.78
$60 to <$100 K	14	8.2 (3.4–13.1)	213	9.6 (8.1–11.0)	27.0 (23.7–30.4)	22.9 (21.7–24.0)	**0**	**0.35 (0.16–0.55)**	0.00
≥$100 K	8	3.9 (0.8–6.9)	100	4.6 (3.5–5.6)	27.4 (19.7–31.6)	25.3 (23.8–26.8)	**0**	**0.29 (0.08–0.50)**	0.00
**Dental insurance**									
No	152	**63.9 (56.7–71.1)**	1524	**54.2 (52.0–56.4)**	20.3 (18.6–21.9)	18.3 (17.7–18.8)	0.97 (0.54–1.40)	0.95 (0.76–1.14)	1.02
Yes	93	**36.1 (28.9–43.3)**	1321	**45.8 (43.6–48.0)**	21.5 (19.1–23.9)	21.0 (20.5–21.5)	**0.07 (0.0–0.16)**	**0.44 (0.31–0.59)**	0.16
**Last dental visit**									
12+ months ago	83	34.0 (26.8–41.2)	931	32.5 (30.5–34.6)	18.3 (16.0–20.6)	16.8 (16.1–17.5)	1.16 (0.63–1.70)	1.38 (1.06–1.63)	0.84
<12 months ago	162	66.0 (58.8–73.2)	1920	67.5 (65.4–69.5)	22.0 (20.4–23.7)	20.8 (20.4–21.3)	0.47 (0.08–0.86)	0.40 (0.32–0.49)	1.18
**Country of birth**									
Oversea	226	**93.1 (89.4–96.8)**	638	**21.6 (19.8–23.4)**	20.6 (19.2–22.0)	20.5 (19.8–21.2)	0.81 (0.47–1.16)	1.04 (0.72–1.37)	0.78
Australia	19	**6.9 (3.2–10.6)**	2215	**78.4 (76.6–80.2)**	20.1 (15.4–24.8)	19.3 (18.8–19.7)	**0.05 (0.00–0.23)**	**0.62 (0.50–0.74)**	0.08
	**2017–2018 (n = 4641)**								
	**Number, weighted % (95% CI)**				**Mean number of teeth (95%CI)**		**Mean DS (95% CI)**		Ratio
	CALD		Non-CALD		CALD	Non-CALD	CALD	Non-CALD	
**Total**	363	12.8 (11.3–14.4)	4278	87.2 (85.6–88.7)	21.9 (20.6–23.2)	21.2 (20.8–21.5)	1.42 (0.91–1.93)	1.50 (1.23–1.77)	0.95
**Age group (years)**									
60–69	198	55.2 (48.7–61.7)	2134	50.9 (49.0–52.8)	24.0 (22.6–25.4)	22.6 (22.2–23.1)	1.38 (0.56–2.21)	2.00 (1.58–2.43)	0.95
70–79	117	30.2 (24.4–36.0)	1607	36.9 (35.1–38.8)	21.3 (19.1–23.6)	19.8 (19.2–20.3)	1.43 (0.60–2.26)	0.87 (0.62–1.12)	1.64
≥ 80	48	14.6 (9.8–19.4)	537	12.2 (11.0–13.4)	13.6 (8.8–18.4)	18.8 (17.8–19.8)	2.64 (1.14–4.15)	2.14 (1.06–3.22)	1.23
**Sex**									
Male	162	43.7 (37.2–50.2)	2020	48.6 (46.7–50.5)	20.4 (20.7–24.0)	21.1 (20.6–21.6)	1.88 (1.05–2.72)	2.20 (1.72–2.68)	0.85
Female	201	56.3 (49.8–62.8)	2258	51.4 (49.5–53.3)	21.2 (19.0–23.4)	21.2 (20.7–21.7)	0.72 (0.32–1.12)	0.77 (0.57–0.98)	0.94
**Residential location**									
Regional/remote	60	**9.3 (6.2–12.3)**	1870	**35.8 (34.0–37.6)**	22.4 (19.0–25.9)	20.5 (20.0–21.1)	1.49 (0.93–2.10)	2.01 (1.24–2.11)	0.74
Major city	303	**90.7 (87.7–93.8)**	2408	**64.2 (62.4–66.0)**	21.9 (20.4–23.3)	21.5 (21.0–21.9)	**0.46 (0.0–1.10)**	**1.30 (1.13–1.85)**	0.35
**Household income**									
<$30 K	47	21.0 (14.3–27.8)	430	13.3 (11.8–14.8)	23.8 (21.3–26.4)	21.7 (20.7–22.7)	2.54 (0.58–4.50)	2.43 (1.17–3.69)	1.05
$30 K to <$60 K	156	55.7 (47.7–63.6)	1890	54.3 (52.1–56.4)	19.9 (17.6–22.1)	20.2 (19.7–20.7)	1.21 (0.72–1.69)	1.61 (1.20–2.01)	0.75
$60 to <$100 K	35	15.1 (8.9–21.3)	661	18.4 (16.7–20.0)	**26.1 (24.5–27.8)**	**23.1 (22.4–23.8)**	0.45 (0.0–1.24)	1.38 (0.85–1.91)	0.33
≥$100 K	29	**8.2 (4.5–11.9)**	532	**14.1 (12.6–15.5)**	21.3 (16.5–26.1)	23.6 (22.7–24.6)	**0**	**0.68 (0.42–0.94)**	0
**Dental insurance**									
No	218	**67.4 (61.6–73.3)**	1883	**46.6 (44.7–48.5)**	21.5 (19.7–23.2)	19.7 (19.2–20.2)	1.60 (0.94–2.26)	2.12 (1.62–2.61)	0.75
Yes	143	**32.6 (26.7–38.4)**	2364	**53.4 (51.5–55.3)**	23.2 (21.4–25.1)	22.8 (22.4–23.2)	0.98 (0.14–1.81)	0.88 (0.65–1.10)	1.11
**Last dental visit**									
12+ months ago	151	**48.6 (41.9–55.2)**	1393	**34.0 (32.2–35.9)**	21.6 (19.2–23.9)	20.1 (19.5–20.7)	1.54 (0.84–2.25)	2.70 (2.02–3.37)	0.57
<12 months ago	208	**51.4 (44.8–58.1)**	2865	**66.0 (64.1–67.8)**	22.3 (21.0–23.6)	21.7 (21.3–22.1)	1.27 (0.51–2.04)	0.69 (0.57–0.82)	1.84
**Country of birth**									
Oversea	325	**93.3 (90.5–96.0)**	1076	**31.3 (29.4–33.2)**	21.7 (20.3–23.1)	22.2 (21.5–22.8)	1.15 (0.78–1.52)	1.10 (0,69–1.52)	1.05
Australia	38	**6.7 (4.0–9.5)**	3201	**68.7 (66.8–70.6)**	24.8 (20.9–28.7)	20.7 (20.3–21.1)	**7.00 (2.57–11.43)**	**1.77 (1.45–2.10)**	3.95

**Notes**: ^a^: CALD/non-CALD; Bold were donated as statistically significant.

**Table 2:** Decomposition of the change in mean DS among CALD and non-CALD Australian adults aged ≥60 years between 2004–2006 and 2017–2018.

	2004–2006				2017–2018			
	CALD Aged ≥60 Years				CALD Aged ≥60 Years			
Mean DS (Regional/remote)	1.395				1.493			
Mean DS (Major city)	0.647				0.456			
Due to endowments (E)	0.460				0.782			
Due to coefficients (C)	−0.100				1.356			
Due to interaction (CE)	0.388				−1.101			
Explained %	61.6				75.4			
Unexplained %	38.4				24.6			
Explanatory Variables	E	C	E (Neumark ^a^)	Proportion explained (%)	E	C	E (Neumark ^a^)	Proportion explained (%)
Age group	0.002	23.17	−0.004	−0.95	0.330	13.437	−0.040	10.07
Sex	0.016	0.499	0.072	15,63	0.160	1.209	0.026	15.81
Household income	0.044	−7.369	0.037	8.04	0.268	0.721	0.197	80.08
Dental insurance	0.168	3.763	0.159	* 38.22	0.004	−1.090	−0.009	−3.77
Last dental visit	0.222	3.169	0.146	34.40	0.039	0.045	0.039	15.88
Country of birth	0.007	−2.145	0.018	4.67	−0.020	2.428	−0.045	−18.06
Total	0.460	−0.100	0.428	100.00	0.782	1.356	−0.168	100
	Non-CALD aged ≥60 years				Non-CALD aged ≥60 years			
Mean DS (Regional/remote)	0.815				2.006			
Mean DS (Major city)	0.646				1.300			
Due to endowments (E)	0.131				0.459			
Due to coefficients (C)	0.058				−0.089			
Due to interaction (CE)	−0.020				0.335			
Explained %	77.7				65.1			
Unexplained %	22.3				34.9			
Explanatory variables	E	C	E (Neumark ^a^)	Proportion explained (%)	E	C	E (Neumark ^a^)	Proportion explained (%)
Age group	0.011	1.408	0.005	4.30	0.012	−2.837	0.027	1.22
Sex	0.005	−0.118	0.006	4.61	0.097	−2.180	0.168	* 27.31
Household income	0.035	−0.112	0.040	* 32.53	0.047	1.517	0.030	4.59
Dental insurance	0.018	−0.136	0.023	18.24	0.011	−2.655	0.059	9.96
Last dental visit	0.009	0.381	0.089	** 72.86	0.198	−1.231	0.246	* 42.54
Country of birth	−0.037	−0.075	−0.039	−32.54	0.093	−0.732	0.088	14.38
**Total**	0.131	0.058	0.124	100.00	0.459	−0.089	0.614	100.00

** *p*-value < 0.01; * *p*-value < 0.05. ^a^ (Neumark, 1988) [18] the coefficients obtained from the pooled data regression. The coefficients were obtained from the pooled data regression. E (and E Neumark), C, and CE show the contribution attributable to the gaps in endowments (E), the coefficients (C), and due to the interaction (CE). In this study, the gap in endowments accounts for the great bulk of the gap in outcomes. Proportion explained: related to change in endowments, attributable to residential location level changes in the magnitude of the explanatory variables. Unexplained: related to change in coefficients. Untreated dental caries surfaces for primary dentition.

## Data Availability

The datasets generated and/or analysed during the current study are not publicly available due to privacy issues of the participants. Data are available from the corresponding author on reasonable request.
